# Time trends in facility-based and private-sector childbirth care: analysis of Demographic and Health Surveys from 25 sub-Saharan African countries from 2000 to 2016

**DOI:** 10.7189/jogh.09.020406

**Published:** 2019-12

**Authors:** Henry Victor Doctor, Emma Radovich, Lenka Benova

**Affiliations:** 1 World Health Organization, Regional Office for the Eastern Mediterranean, Cairo, Egypt; 2 Faculty of Epidemiology and Population Health, London School of Hygiene & Tropical Medicine, London, UK; 3 Department of Public Health, Institute of Tropical Medicine, Antwerp, Belgium

## Abstract

**Background:**

Africa, and sub-Saharan Africa in particular, remains one of the regions with modest improvements to maternal and newborn survival and morbidity. Good quality intrapartum and early postpartum care in a health facility as well as delivery under the supervision of trained personnel is associated with improved maternal and newborn health outcomes and decreased mortality. We describe and contrast recent time trends in the scale and socio-economic inequalities in facility-based and private facility-based childbirth in sub-Saharan Africa.

**Methods:**

We used Demographic and Health Surveys in two time periods (2000-2007 and 2008-2016) to analyse levels and time trends in facility-based and private facility-based deliveries for all live births in the five-year survey recall period to women aged 15-49. Household wealth quintiles were used for equity analysis. Absolute numbers of births by facility sector were calculated applying UN Population Division crude birth rates to the total country population.

**Results:**

The percentage of all live births occurring in health facilities varied across countries (5%-85%) in 2000-2007. In 2008-2016, this ranged from 22% to 92%. The lowest percentage of all births occurring in private facilities in 2000-2007 period was in Ethiopia (0.3%) and the highest in the Democratic Republic of Congo at 20.5%. By 2008-2016, this ranged from 0.6% in Niger to 22.3% in Gabon. Overall, the growth in the absolute numbers of births in facilities outpaced the growth in the percentage of births in facilities. The largest increases in absolute numbers of births occurred in public sector facilities in all countries. Overall, the percentage of births occurring in facilities was significantly lower for poorest compared to wealthiest women. As the percentage of facility births increased in all countries over time, the extent of wealth-based differences had reduced between the two time periods in most countries (median risk ratio in 2008-2016 was 2.02). The majority of countries saw a narrowing in both the absolute and relative difference in facility-based deliveries between poorest and wealthiest.

**Conclusions:**

The growth in facility-based deliveries, which was largely driven by the public sector, calls for increased investments in effective interventions to improve service delivery and quality of life for the mother and newborn. The goal of universal health coverage to provide better quality services can be achieved by deploying interventions that are holistic in managing and regulating the private sector to enhance performance of the health care system in its entirety rather than interventions that only target service delivery in one sector.

About 830 women die from complications related to pregnancy or childbirth globally every day [1]; most of these deaths can be prevented or treated. Africa, and sub-Saharan Africa in particular, remains one of the regions with modest improvements to maternal and newborn survival and morbidity [2]. The region is largely characterized by high maternal mortality ratios (MMRs) and perinatal mortality. Globally, MMR declined by an average of 3.0% annually between 2000 and 2015, which was more than double the estimated average annual decline of between 1990 and 2000 (1.2%). While estimated MMRs declined across all regions between 1990 and 2015, the magnitude of these reduction varied significantly between regions; with sub-Saharan Africa declining by 45% compared with Eastern Asia at 72%. Within sub-Saharan Africa, there were large across-country variations in MMR time trends; including large declines in Eastern African countries (57%) compared to an increase of 4% in Southern African countries [3].

Evidence shows that good quality intrapartum and early postpartum care in a health facility as well as delivery under the supervision of trained personnel are associated with improved maternal and newborn health outcomes and decreased mortality [4-6]. The association between childbirth in a health facility and improved maternal and newborn health outcomes has been documented [7-9]. Women who deliver in health facilities which provide basic and comprehensive emergency obstetric care are more likely to have improved health outcomes than women who deliver outside the health facility. However, availability, quality, accessibility, and utilization of well-equipped facilities in sub-Saharan Africa continue to be a challenge. This is largely influenced by limited investments in health service delivery, socio-cultural factors, lack of understanding on the benefits of skilled attendance at birth, financial hardship and physical accessibility, among others [10,11]. Moreover, other studies [12] have found that facility deliveries can, though not necessarily, lower early neonatal mortality; suggesting the importance of the context and quality in which facility deliveries occur.

Despite these challenges, a recent study which analysed data from 58 Demographic and Health Surveys (DHS) collected between 1990 and 2015 in 29 sub-Saharan African countries found an overall increase in facility-based deliveries in later surveys (conducted since 2010) compared to surveys conducted since the 1990s [13]. Specifically, births from more recent surveys were 85% more likely to occur in facilities than births from earliest surveys, but with wide variations across countries. While the findings of this study demonstrated some progress in the coverage of facility deliveries, it was found that the overall proportion of births occurring in facilities was still too low to achieve significant gains towards universal health coverage; and varied by country and sub-Saharan African sub-region.

While previous studies documented recent trends in and determinants of health facility delivery in sub-Saharan Africa [13-16], there are still knowledge gaps about trends in private facility births and the contribution of private health care facilities to the observed trends in health facility delivery. A paper highlighted the extent of private sector use for childbirth care across low- and middle-income countries (LMIC), and estimated that 10% of childbirth in sub-Saharan Africa took place in the private sector [17]. This paper did not assess trends over time, or how the two sectors differentially contribute to the overall increase in facility delivery [17]. Additionally, a gap in the literature exists in describing, as comprehensively as possible from available data, the pattern seen across countries in sub-Saharan Africa. In the 1990s, many governments in sub-Saharan Africa began to focus on increasing engagement with private health care providers [18,19]. The engagements were made within the context of structural adjustments in which governments were required to reduce public spending on health [20,21]. While use of private delivery care has been on the rise, there are concerns that private delivery care may have adverse effects on maternal and child health, including equity and quality of care due to a plethora of private health care services that may not be properly regulated [22]. Another concern relates to the diversity of private health providers, including different profit, ownership and governance models and structures of care that may lead to lack of accountability in provision of evidence-based care and exacerbate inequities in access to care [23].

The rapid and extensive privatization initiatives have been associated with poor quality of delivery care in the private sector than the public health sector [23]. However, there is a growing need to know how the private sector services compare to those of the public sector to inform evidence based decision making. Other studies that have responded to this gap by comparing the quality of formal private vs public ambulatory health care in LMICs found that drug supply, responsiveness and service delivery effort was better in the private sector than in the public sector [24]. These findings demonstrate some of the existing challenges associated with health service delivery between the public and the private sector that may have implications on the observed trends in health facility delivery. Within the context of the Sustainable Development Goal (SDG) on health (SDG 3), many sub-Saharan African countries also continue in their efforts to strengthen health system delivery to meet the target of lowering MMRs to under 70 deaths per 100 000 live births by 2030. The “conceptualisation, systematic measurement, and effective tackling of coverage and configuration challenges to implement high quality, respectful maternal health care” are key outstanding issues to addressing preventable maternal and newborn mortality [25]. Within this large umbrella, understanding trends in use and equity of coverage of the private sector for delivery care can inform strategies for governments’ engagement with private providers [26].

The objective of this paper is to describe and contrast time trends over the period of the Millennium Development Goals (MDGs) – a period of significant investment in maternal health [27] – i) in the scale of, and ii) socio-economic inequalities in facility-based and private facility-based childbirth in sub-Saharan Africa. We acknowledge the wide variation in the health systems in sub-Saharan Africa, including the contributions and compositions of the public and private sectors. Based on this variation, we decided not to pool the available data from the included countries, but rather focus on describing the changes over time for each country separately, to retain and assess the extent of this variability. The comparison is 2-fold: within-country over time and across countries in the region. Our selection of data points (surveys available in the intervals specified) was partly guided by the highest number of countries that could be included, to extend the second comparison.

## Methods

### Data and population

DHS are cross-sectional nationally-representative household surveys which have been regularly collected in many countries of sub-Saharan Africa for several decades. Each country uses a standard model questionnaire that can be adjusted to address country specific needs, but resulting data sets are standardized to enable comparison across countries. Typical survey questions collect responses about household characteristics and women of reproductive age (15-49 years). The DHS use multi-level cluster sampling design allowing for oversampling in certain areas.

To assess time trends in facility deliveries since 2000, we included all sub-Saharan African countries which conducted at least one survey in both time periods: 2000-2007 (8 years) and 2008-2016 (9 years); 25 countries met these criteria (Appendix S1 in **Online Supplementary Document**). If more than one survey was available in either time period, we included the earliest in the 2000-2007 and the most recent in the 2008-2016 period. These time periods were selected to span the period of the MDGs, to maximise the number of countries meeting the inclusion criteria, and to avoid overlap in the survey recall periods. Thus, no country included in the analysis has an overlapping survey recall period since the gap between surveys included is more than 5 years. We used the children ever born section of the women’s questionnaire; all live births in the five-year survey recall period to women aged 15-49 at the time of the survey were included in the analysis.

### Data analysis

We assessed the location of delivery based on women’s responses to the question “Where did you give birth to (Name of Child)?” Locations were categorized into home vs facility-based, and among facility-based deliveries, into public vs private sector according to previously published categorisation [17]. The private sector includes non-public organizations of various types, such as for-profit, faith-based organizations and non-governmental organizations. We disaggregated by household wealth quintile [28].

Analysis was conducted using Stata 15 SE [29] and adjusting for survey design according to DHS instructions (ie, using *svyset* command for clustering, survey weights, and stratification) to produce point estimates and associated 95% confidence intervals. We estimated the percentage of all births occurring in facilities and within facilities in each sector (public facility births + private facility births = all facility births). Findings from each country are presented separately; no pooled analysis was conducted due to contextual heterogeneity. Trends over time in each country were expressed as a compound annual growth rate between surveys at time 1 and time 2, for the number of years elapsed between the two surveys, similar to an earlier study [30]. Absolute numbers of births by facility sector were calculated by multiplying the proportions in each time period to estimates of the total number of births, which were obtained by applying United Nations Population Division [31] crude birth rates to the total country population in the calendar year two years before the year of the survey to approximate the mid-point of the surveys’ five year recall period. (eg, 1998 for a 2000 survey). To assess equity, we compared poorest and wealthiest household wealth quintiles.

Missing data in the variable capturing location of delivery was very low. We dropped observations with a system missing (“.”) in location of delivery from our analyses (1950 of 481 732 live births in analysis – 0.4%). We categorised the 3883 (0.8%) births that were reported in a “Missing/Don’t know” and “Other” location as occurring in home settings. There were no missing data in the variable capturing household wealth quintile.

### Ethical review

The DHS receive government permission, use informed consent and assure respondents of confidentiality. The Research Ethics Committee of the London School of Hygiene and Tropical Medicine approved our analyses.

## RESULTS

### Facility births

Among the 25 included countries, the number of years between surveys in time 1 (2000-2007) and time 2 (2008-2016) ranged from six (Liberia and Niger) to 16 (Ethiopia, Malawi and Uganda). Across the surveys at time 1, the percentage of all live births occurring in health facilities was lowest in Ethiopia at 5.0% and highest in Gabon at 84.7%, as shown in **Table 1**. By time 2, the percentage of facility-based ranged from 21.9% in Chad to 91.6% in the Republic of the Congo (Congo), having increased by between 1.9 percentage points (Cameroon) to 64.1 percentage points in Rwanda. The relative increase in percentage of births in facilities, expressed asa risk ratio, was significant at the *P* < 0.05 level in all countries except in Cameroon, Mozambique and Nigeria. The compound annual growth rates ranged from 0.5% (Cameroon and Gabon) to 10.9% in Ethiopia, with a median of 3.2% across the countries.

**Table 1:** Percent of all live births in survey recall period occurring in health facilities, by time of survey

Country	No. of years between surveys	T1 (2000-2007)		T2 (2008-2016)		Absolute percentage point difference (T2-T1)	Compound annual growth rate (T1->T2)	Risk Ratio (T2/T1)	Risk ratio *P*-value
**Estimate**	**95% CI**	**Estimate**	**95% CI**
Benin	10	76.4%	72.3-80.1	87.2%	85.2-88.9	10.7%	1.3%	1.14	<0.001
Burkina Faso	7	38.5%	34.7-42.5	66.5%	63.5-69.4	28.0%	8.1%	1.73	<0.001
Cameroon	7	59.3%	54.9-63.5	61.2%	57.0-65.2	1.9%	0.5%	1.03	0.544
Chad	11	13.2%	10.4-16.7	21.9%	19.7-24.2	8.6%	4.7%	1.65	<0.001
Rep of the Congo	7	82.2%	77.5-86.1	91.6%	89.6-93.1	9.4%	1.6%	1.11	<0.001
Dem Rep of Congo	7	71.0%	66.0-75.6	80.2%	77.2-83.0	9.2%	1.8%	1.13	0.001
Ethiopia	16	5.0%	4.0-6.3	26.2%	23.1-29.6	21.2%	10.9%	5.22	<0.001
Gabon	12	84.7%	82.2-86.9	90.2%	88.3-92.0	5.6%	0.5%	1.07	<0.001
Ghana	11	45.9%	42.1-49.8	73.1%	69.5-76.4	27.2%	4.3%	1.59	<0.001
Guinea	7	30.9%	27.2-35.0	40.6%	36.6-44.7	9.6%	3.9%	1.31	0.001
Kenya	11	40.2%	37.1-43.4	61.5%	59.6-63.3	21.2%	3.9%	1.53	<0.001
Lesotho	10	53.0%	50.3-55.6	76.5%	74.0-78.8	23.6%	3.7%	1.44	<0.001
Liberia	6	37.4%	32.9-42.1	55.9%	52.1-59.7	18.6%	6.9%	1.50	<0.001
Malawi	16	55.5%	52.9-58.1	91.4%	90.4-92.4	35.9%	3.2%	1.65	<0.001
Mali	12	38.1%	34.0-42.5	55.0%	51.2-58.8	16.9%	3.1%	1.44	<0.001
Mozambique	8	50.8%	47.5-54.1	54.8%	51.4-58.1	4.0%	1.0%	1.08	0.072
Namibia	13	75.5%	71.5-79.0	87.7%	85.9-89.4	12.3%	1.2%	1.16	<0.001
Niger	6	17.3%	14.9-20.1	30.0%	27.3-32.8	12.6%	9.6%	1.73	<0.001
Nigeria	10	32.8%	28.4-37.6	36.2%	33.6-38.8	3.4%	1.0%	1.10	0.160
Rwanda	15	26.6%	24.3-29.1	90.7%	89.5-91.8	64.1%	8.5%	3.41	<0.001
Senegal	10	62.2%	58.2-65.9	74.5%	70.4-78.2	12.4%	1.8%	1.20	<0.001
Tanzania	11	47.2%	43.9-50.4	62.6%	59.4-65.7	15.4%	2.6%	1.33	<0.001
Uganda	16	36.8%	33.3-40.5	73.4%	71.5-75.2	36.6%	4.4%	1.99	<0.001
Zambia	13	43.8%	40.0-47.7	67.6%	65.2-70.0	23.9%	3.4%	1.55	<0.001
Zimbabwe	10	68.0%	64.8-71.0	77.0%	74.1-79.6	9.0%	1.2%	1.13	<0.001

### Private facility births

**Table 2** shows that among surveys at time 1, the lowest percentage of all births occurring in private facilities was in Ethiopia (0.3%) and the highest in the Democratic Republic of Congo (DRC) at 20.5%. By time 2, this ranged from 0.6% in Niger to 22.3% in Gabon. The absolute percentage point difference over the analysis period ranged from a decrease of 5.5 percentage points in the DRC to an increase of 6.9 percentage points in Gabon. The highest compound annual rate of decrease in private facility births was in Rwanda (-7.1%); with the highest annual rate of increase in Guinea (11.7%); the median across countries was 0.7%. The relative changes over time, expressed by risk ratios, show that there was no significant change in the percentage of births occurring in private health facilities in 12, a significant increase in seven, and a significant decrease in the remaining six countries.

**Table 2:** Percent of all live births in survey recall period occurring in private facilities, by time of survey

Country	No. of years between surveys	T1 (2000-2007)		T2 (2008-2016)		Absolute percentage point difference (T2-T1)	Compound annual growth rate (T1->T2)	Risk Ratio (T2/T1)	Risk ratio *P* value
**Estimate**	**95% CI**	**Estimate**	**95% CI**
Benin	10	11.3%	9.5-13.4	12.0%	10.7-13.4	0.7%	0.6%	1.06	0.530
Burkina Faso	7	0.8%	0.5-1.4	1.0%	0.6-1.6	0.2%	2.5%	1.19	0.581
Cameroon	7	17.8%	15.6-20.2	21.0%	18.8-23.4	3.3%	2.4%	1.18	0.054
Chad	11	2.0%	1.2-3.2	1.1%	0.8-1.5	-0.9%	-5.1%	0.56	0.041
Rep of the Congo	7	7.2%	5.8-8.8	11.6%	9.6-14.0	4.4%	7.1%	1.62	<0.001
Dem Rep of Congo	7	20.5%	16.7-24.8	15.4%	12.8-18.5	-5.0%	-4.0%	0.75	0.022
Ethiopia	16	0.3%	0.2-0.5	1.4%	1.1-1.9	1.1%	9.5%	4.24	<0.001
Gabon	12	15.4%	13.3- 17.7	22.3%	19.1-25.8	6.9%	3.2%	1.45	<0.001
Ghana	11	9.4%	7.9-11.2	8.1%	6.7-9.7	-1.3%	-1.4%	0.86	0.233
Guinea	7	1.5%	1.0-2.2	4.7%	3.6-6.1	3.2%	17.7%	3.13	<0.001
Kenya	11	14.0%	12.3-15.9	15.2%	14.0-16.6	1.2%	0.8%	1.09	0.278
Lesotho	10	14.5%	12.8-16.5	18.3%	16.0-20.9	3.8%	2.3%	1.26	0.015
Liberia	6	10.1%	8.2-12.3	12.5%	9.6-16.0	2.4%	3.6%	1.24	0.181
Malawi	16	15.2%	13.9-17.2	12.7%	11.2-14.4	-2.5%	-1.1%	0.83	0.048
Mali	12	0.9%	0.7- 1.3	2.4%	1.8-3.2	1.5%	8.1%	2.55	<0.001
Mozambique	8	3.2%	2.6-4.0	2.1%	1.8-2.5	-1.1%	-5.1%	0.66	0.002
Namibia	13	4.9%	2.8-8.3	5.2%	4.0-6.7	0.3%	0.5%	1.07	0.820
Niger	6	0.4%	0.3-0.7	0.6%	0.5-0.8	0.2%	5.3%	1.36	0.210
Nigeria	10	14.5%	11.9-18.5	13.4%	11.8-15.1	-1.1%	-0.8%	0.92	0.538
Rwanda	15	2.3%	1.9-2.8	0.8%	0.5-1.1	-1.5%	-7.1%	0.33	<0.001
Senegal	10	4.0%	2.9-5.6	3.7%	2.6-5.2	-0.3%	-0.9%	0.92	0.721
Tanzania	11	9.3%	7.7-11.2	12.0%	10.5-13.6	2.7%	2.3%	1.29	0.028
Uganda	16	14.5%	12.5-16.6	16.1%	14.7-17.6	1.6%	0.7%	1.11	0.191
Zambia	13	9.1%	7.0-11.8	4.8%	3.9-5.9	-4.4%	-4.9%	0.52	<0.001
Zimbabwe	10	12.5%	10.7-14.6	11.9%	10.0-14.3	-0.6%	-0.5%	0.95	0.689

### Number of births

**Figure 1:**
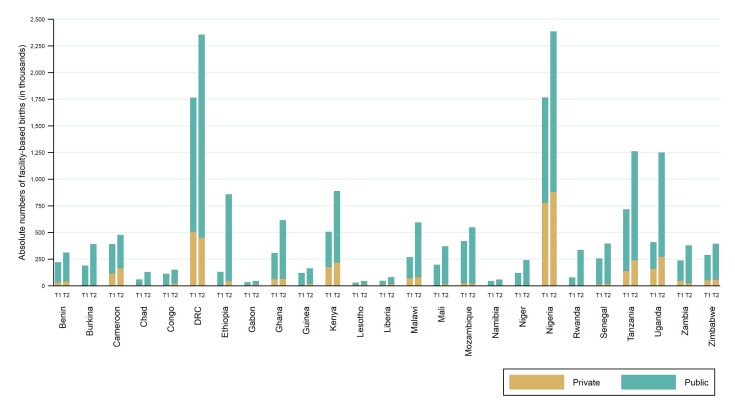
Absolute annual number of facility-based births between T1 and T2 by sector of facility.

**Figure 1** shows the absolute annual numbers of facility-based births by sector in time periods 1 and 2 for each country (absolute differences and relative change over time in numbers of births are presented in Appendix S2 in **Online Supplementary Document**). At time 1, the absolute number of annual births in facilities ranged from around 30 000 births in Lesotho to nearly 1.8 million in Nigeria. There was an increase in the total number of facility births in all countries between the two time periods, with the number of additional annual births in facilities ranging from 12 000 in Gabon to 840 000 in Uganda. The relative increase over time in the number of facility-based births ranged from 22.1% in Cameroon to 558.1% in Ethiopia. The growth in the numbers of births in facilities outpaced the growth in the proportion of facility-based deliveries in every country.

The largest increases in absolute numbers of births occurred in public sector facilities in all countries. In four countries (DRC, Ethiopia, Nigeria and Uganda), facilities in the public sector accommodated over half a million additional annual births between surveys in time 1 and 2. The absolute numbers of births in private facilities at time 1 ranged from fewer than 3000 in Namibia to nearly 800 000 in Nigeria. The relative changes in the number of private facility births over time ranged from a decrease of 58.7% in Rwanda to an increase of 435.0% in Ethiopia. The annual compound growth rate ranged from a decrease of 5.7% to an increase of 18.4%; with a median across countries of +2.8%.

### Facility births by wealth quintile

We used the DHS wealth quintiles to estimate inequalities in the percentages of all births in facilities and in private facilities between women from the poorest and wealthiest quintiles of households. The results (**Table 3**) show that in both time periods, the percentage of births occurring in facilities was significantly lower for poorest compared to wealthiest women. At time 1, the percentage of births among wealthiest women was between 1.51 (Congo) and 37.1 (Chad) times higher compared to poorest; the median risk ratio was 3.6 across the countries. As the percentage of facility births increased in all countries over time, the extent of wealth-based differences had reduced between the two time periods in most countries (median risk ratio at time 2 was 2.02). That is, the percentage (or likelihood) of births among women in wealthiest households that occurred in health facilities was more than twice the percentage of births among women in poorest households.

**Table 3:** Percent of all live births in survey recall period occurring in health facilities, by time of survey and wealth quintile*

Country	T1				T2			
**Wealthiest**	**Poorest**	**Absolute difference (W-P)**	**Risk Ratio (W/P)**	**Wealthiest**	**Poorest**	**Absolute difference (W-P)**	**Risk Ratio (W/P)**
Benin	98.3%	55.5%	42.8%	1.77	99.3%	70.9%	28.4%	1.40
Burkina Faso	84.6%	19.6%	65.0%	4.32	93.5%	46.2%	47.3%	2.02
Cameroon	91.7%	27.7%	64.0%	3.31	95.0%	17.1%	77.9%	5.56
Chad	42.6%	1.2%	41.5%	37.08	57.8%	13.0%	44.7%	4.43
Rep of the Congo	97.1%	64.4%	32.7%	1.51	99.2%	75.3%	23.9%	1.32
Dem Rep of Congo	97.6%	55.8%	41.8%	1.75	97.9%	65.8%	32.1%	1.49
Ethiopia	22.8%	0.7%	22.1%	33.53	68.6%	10.6%	58.1%	6.48
Gabon	96.7%	63.9%	32.8%	1.51	96.3%	75.5%	20.9%	1.28
Ghana	90.7%	19.5%	71.2%	4.65	96.6%	46.0%	50.6%	2.10
Guinea	70.1%	11.6%	58.5%	6.04	81.5%	17.7%	63.8%	4.60
Kenya	74.0%	16.1%	57.9%	4.61	92.9%	30.3%	62.6%	3.07
Lesotho	81.7%	30.6%	51.1%	2.67	92.9%	56.9%	36.0%	1.63
Liberia	70.9%	18.4%	52.5%	3.86	79.4%	40.6%	38.7%	1.95
Malawi	83.0%	42.9%	40.1%	1.94	96.4%	88.6%	7.8%	1.09
Mali	85.0%	20.2%	64.7%	4.20	93.6%	27.7%	65.9%	3.38
Mozambique	90.1%	30.6%	59.6%	2.95	91.6%	31.2%	60.4%	2.93
Namibia	96.6%	54.5%	42.2%	1.77	98.4%	71.4%	27.0%	1.38
Niger	58.4%	4.9%	53.5%	12.02	71.5%	12.9%	58.6%	5.53
Nigeria	79.9%	11.6%	68.3%	6.88	80.7%	5.9%	74.8%	13.69
Rwanda	59.3%	16.4%	42.9%	3.62	97.1%	84.2%	12.9%	1.15
Senegal	94.3%	29.0%	65.2%	3.25	95.3%	44.4%	50.9%	2.15
Tanzania	86.7%	32.1%	54.6%	2.70	94.4%	40.9%	53.4%	2.31
Uganda	76.5%	18.5%	58.0%	4.13	92.7%	64.2%	28.5%	1.44
Zambia	91.5%	20.1%	71.3%	4.55	95.4%	49.7%	45.8%	1.92
Zimbabwe	94.7%	45.7%	49.0%	2.07	95.2%	60.8%	34.4%	1.57

W – wealthiest quintile; P – poorest quintile.

*The *P*-values for differences in risk ratios between the wealthiest and poorest households were all <0.001.

**Figure 2:**
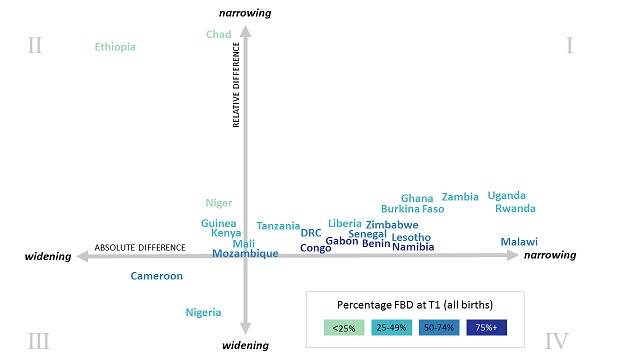
Typologies of change in absolute and relative difference of faculty-based deliveries (FBD) between wealthiest and poorest quintiles from T1 (2000-2007) to T2 (2008-2016), chart not to scale.

We characterised the patterns of inequality over time by whether the relative and absolute differences in the percentage of facility-based deliveries are narrowing or widening between the poorest and wealthiest quintile. **Figure 2** shows these typologies of change - the majority of countries saw a narrowing in both the absolute and relative difference between poorest and wealthiest, shown in quadrant 1 (I) of the figure. As these countries moved toward near-universal facility-based delivery rates among women from the wealthiest quintile of households, the absolute wealth-based differences narrowed in a greater extent compared to relative differences. Notably, the three countries in which less than 25% of births occurred in facilities at time 1 (Niger, Ethiopia, and Chad) saw a widening in absolute wealth-based differences, yet a narrowing in the relative differences (II). This reflects gains primarily among the wealthy in a situation where women from both wealthiest and poorest quintiles are increasingly using facilities for deliveries, and where the level of facility-based delivery among the wealthiest is far below universal. In Cameroon and Nigeria (III), both relative and absolute wealth-based differences were widening; this is due to the decline between time 1 and 2 in the percentage of poorest women giving births in facilities.

**Figure 3:**
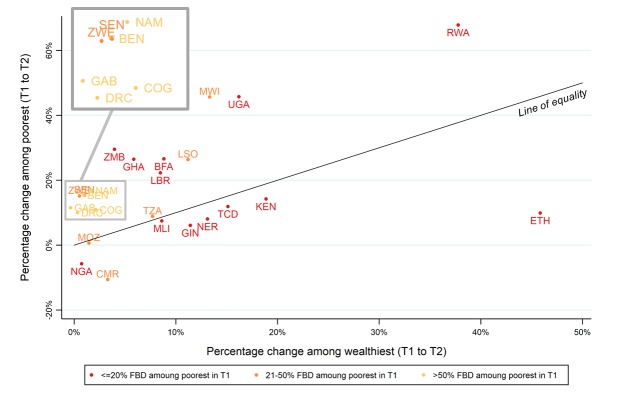
Percentage change in faculty-based deliveries (FBD) (T1 to T2) among wealthiest and poorest quintiles.

**Figure 3** shows the comparison of trends in facility-based deliveries between time 1 and 2 by wealth. Countries above the diagonal line of equality had a faster rate of growth among women from the poorest than richest quintile (15 of the 25 countries). The colours of the labels indicate three levels of the percentage of women in the poorest quintile giving birth in facilities at time 1. Notably, both Rwanda and Ethiopia started at very low levels at time 1, but show different pathways of development to time 2. A larger increase among poorest women compared to wealthiest was seen in Rwanda (above the line of equality), and the opposite phenomenon occurred in Ethiopia (below the line of equality).

### Private facility births by wealth quintile

**Table 4** shows the trend over time in wealth-based differences in the percentage of live births occurring in private facilities. At time 1, there were seven countries in which less than 1% of births to women from the poorest quintile occurred in private facilities; this number increased to nine countries in time 2. At time 1, Zimbabwe and Malawi had the highest utilisation of private facilities among poorest women, at just over 10%. At time 2, Lesotho had the highest level among poorest women, at 17.3%. The difference between the percentage of deliveries in the private sector between poorest and wealthiest women was significant in the majority of countries in both time periods.

**Table 4:** Percent of all live births in survey recall period occurring in private facilities, by time of survey and wealth quintile

Country	T1					T2				
**Wealthiest**	**Poorest**	**Absolute difference (W-P)**	**Risk Ratio (W/P)**	**Risk ratio *P* value**	**Wealthiest**	**Poorest**	**Absolute difference (W-P)**	**Risk Ratio (W/P)**	**Risk ratio *P*-value**
Benin	31.4%	5.0%	26.5%	6.35	<0.001	30.5%	3.8%	26.7%	7.99	<0.001
Burkina Faso	4.9%	0.0%	4.9%	-	-	6.0%	0.0%	6.0%	-	-
Cameroon	25.7%	8.3%	17.5%	3.11	<0.001	39.1%	3.0%	36.1%	13.15	<0.001
Chad	2.8%	0.6%	2.2%	4.57	>0.001 and <0.05	4.3%	0.5%	3.8%	9.17	<0.001
Rep of the Congo	8.2%	3.8%	4.4%	2.16	>0.001 and <0.05	10.2%	4.4%	5.9%	2.34	<0.001
Dem Rep of Congo	54.9%	5.1%	49.8%	10.84	<0.001	49.0%	5.0%	44.0%	9.77	<0.001
Ethiopia	1.0%	<0.1%	1.0%	177.59	<0.001	7.8%	0.1%	7.6%	59.69	<0.001
Gabon	34.4%	4.0%	30.4%	8.56	<0.001	42.0%	5.4%	36.6%	7.81	<0.001
Ghana	21.7%	2.4%	19.3%	9.02	<0.001	20.1%	1.6%	18.5%	12.69	<0.001
Guinea	8.8%	0.0%	8.8%	-	-	20.1%	0.3%	19.8%	77.27	<0.001
Kenya	30.4%	6.8%	23.6%	4.46	<0.001	37.6%	2.9%	34.8%	13.11	<0.001
Lesotho	23.2%	6.9%	16.2%	3.34	<0.001	21.2%	17.3%	3.9%	1.23	>0.05
Liberia	30.2%	2.2%	28.0%	13.61	<0.001	30.9%	3.2%	27.8%	9.78	<0.001
Malawi	25.1%	10.5%	14.7%	2.40	<0.001	15.0%	11.3%	3.7%	1.33	>0.001 and <0.05
Mali	3.9%	0.4%	3.5%	9.73	<0.001	7.9%	0.8%	7.1%	9.72	<0.001
Mozambique	1.0%	5.6%	-4.7%	0.17	<0.001	1.8%	2.1%	-0.2%	0.88	>0.05
Namibia	10.4%	2.1%	8.4%	5.07	>0.001 and <0.05	29.4%	0.6%	28.9%	51.61	<0.001
Niger	2.2%	0.1%	2.1%	26.95	<0.001	2.2%	0.3%	1.9%	7.40	<0.001
Nigeria	45.0%	4.1%	40.9%	11.10	<0.001	39.1%	1.2%	37.9%	32.07	<0.001
Rwanda	5.8%	1.2%	4.6%	4.70	<0.001	3.8%	0.0%	3.8%	-	-
Senegal	15.4%	0.2%	15.2%	66.87	<0.001	11.7%	0.3%	11.4%	34.41	<0.001
Tanzania	13.4%	6.5%	6.9%	2.07	>0.001 and <0.05	19.6%	6.9%	12.7%	2.85	<0.001
Uganda	31.5%	7.8%	23.7%	4.05	<0.001	32.3%	8.2%	24.1%	3.93	<0.001
Zambia	23.4%	5.4%	18.0%	4.34	<0.001	7.4%	4.9%	2.5%	1.51	>0.001 and <0.05
Zimbabwe	19.1%	10.6%	8.5%	1.80	>0.001 and <0.05	24.6%	7.7%	16.9%	3.20	<0.001

W – wealthiest quintile; P – poorest quintile.

Time trends within the extremes of wealth across the countries can be categorised into four groups: the percentage of births in the private sector increased among both poorest and wealthiest women (eight countries), increased among the poorest women but decreased or remained the same among the wealthiest (four countries), decreased or remain the same among the poorest but increased among the wealthiest (seven countries), and decreased or remained the same among both poorest and wealthiest women (six countries). Across the countries, the median percent of women from the wealthiest quintile using the private sector was 19.1% at time 1 and 20.1% at time 2. Among women from the poorest quintile, the median was 4.0% at time 1 and 2.9% at time 2.

## DISCUSSION

The increase in recent decades in the proportion of births occurring in health facilities in sub-Saharan Africa has been remarkable. This growth is made up of deliveries occurring both in the public and private health sectors, both of which are important sources of health care in sub-Saharan Africa. Despite recent recommendations that African governments should commit at least 15% of their national budget to the health care sector for effective provision of services [32], the burden of health care expenses by households remains high [33]. Although there are several determinants of health care seeking behaviour related to sociocultural factors, perceived benefit/need, economic accessibility and physical accessibility [34], the decision to seek health care from public or private health facility largely depends on the financial resources at the disposal of households. The decision on whether to use private or public health care facilities may also be influenced by claims that the private sector responds more to the needs of patients than the public sector. Others are concerned with the lack of regulation of the private sector and the preponderance of household in paying high out-of-pocket expenses [35].

Our study aimed to understand the contribution of the private sector in the observed increase in facility births by analysing data from 25 sub-Saharan African countries. While we acknowledge the considerable diversity between countries, we found a significant increase in the proportion of facility-based deliveries with the lowest absolute increase observed in Cameroon and highest in Rwanda. Still in time 2 the percent of all live births ranged widely from two in ten women (Chad) to nine in ten women (Congo, Gabon, Malawi, Rwanda). We also showed that the increase in absolute numbers of births in facilities is greater than the increase in the percentage of births in facilities. We found that these additional births were predominantly based in the public sector. In general, in almost all countries, there was an increasing trend in health facility births in the wealthiest quintiles except in Mozambique where the trend had stalled. This stalling may partly be explained by the removal of user fees at the primary care level aimed at increasing use of services among the poor [36]. In 22 countries, the trend in the poorest quintile was also increasing except in Nigeria and Cameroon where it was decreasing; and stalling in Mozambique. In other countries such as Nigeria, the increased home deliveries among the poorest women in areas of high private sector delivery may suggest a preference to avoid public sector facilities where private sector delivery is unaffordable [37].

We found that poor women were less likely to use facility childbirth services and less likely to use private sector facilities for delivery compared to wealthier women, similarly to other studies [14,17,38]. We also found that the highest annual rate of decrease in the percentage of private facility births was in Rwanda (-7.1%); with the highest annual rate of increase in Guinea (11.7%). Rwanda showed greater progress in increasing facility-based childbirth coverage among the poorest compared to the wealthiest between the two time periods.

After characterising the patterns of inequality over time by whether the relative and absolute differences in the percentage of facility-based deliveries were narrowing or widening between the poorest and wealthiest quintile, we found that the majority of countries saw a narrowing in both the absolute and relative difference between poorest and wealthiest. Moreover, the rate of increase in facility-based birth among the poorest was faster than among the richest in all but two countries. A larger increase in facility-based births between time 1 and time 2 among poorest women compared to wealthiest women was observed in Rwanda, and the opposite phenomenon occurred in Ethiopia. The finding of Rwanda is consistent with other reports which have evaluated, among other things, the effectiveness of decentralizing ambulatory reproductive health care services and intrapartum services in increasing antenatal care utilization and skilled attendance at birth in rural Rwandan communities [39].

The finding that facility-based delivery increased over time and led to absolute increases in number of facility births is consistent with other studies [13]. In this paper we explicated that the observed trend in increased facility births was driven predominantly by the public sector. The poor are increasing facility-based deliveries faster and they predominantly use the public sector. The growth of the public health facility-based deliveries may also be influenced by removal of user fees in many countries such as Burkina Faso, Ghana, Liberia, Senegal and Uganda [40,41]; recruitment and training of health care providers [42-44]; and equipping health facilities in countries such as Mozambique and Tanzania [44,45]. Other reasons include disincentives to use traditional birth attendants at home (eg, in Malawi) [46,47]; increased awareness of the benefits of facility-based delivery [13]; and women’s ability to negotiate use of health services [48,49]. Other studies have also reported that for-profit health care may be more appealing due to a number of reasons such as privacy, shorter waiting times, the quest for high quality of care, availability of doctors and as a symbol of status [37]. The increased number of facility births observed since 2000 provides countries with daunting challenges such as strengthening the generally weak health systems and implementing robust health financing structures, particularly in settings where out of pocket health expenditures are high. Increases in health facility births also poses a challenge to the existing health care workforce to deliver high quality services through integrated care [50,51].

### Limitations

The strengths of our paper lie in the inclusion of a large number of countries over a recent time period, using standardized (cross-country comparable) and nationally representative surveys, and using all live births in the surveys’ recall period. However, our results do not include all countries in sub-Saharan Africa because we relied on the availability of a recent DHS. Second, the reliance on women’s recall of events up to five years before the survey and the exclusion of stillbirths (not collected on surveys) might have affected our results. Lastly, the composition of the private sector is not the same across countries and the survey response options do not always capture the organization model under which the private facilities operate (ie, whether they are for profit, faith-based organizations or non-governmental organizations) [52]; hence country-level interpretations require a careful disaggregation of facility types.

## CONCLUSIONS

In sub-Saharan Africa, growth in percentage of births occurring in health facilities since 2000 has been remarkable, especially as this has been achieved despite a substantial growth in the absolute number of births. This growth is driven by the public sector and poor women are more likely to use the public sector. The increases in facility-based deliveries call for investments in effective interventions to improve quality of care. The goal of universal health coverage, which is the core of SDG 3 on health, provides opportunities for countries to design policies that minimizes financial hardships and maximises quality of health care services for all. To achieve this, interventions that are holistic in managing and regulating both the public and private sector are likely to enhance performance of the health care system in both the public and private sector than interventions that only target service delivery in one sector. However, utilisation of facility-based delivery can only address the burden of maternal and perinatal mortality if such care is consistently high quality. Patient safety and quality of care are key elements of universal health coverage. Improving the quality of care and patient safety is one of the key interventions to end preventable maternal and newborn deaths and ensure that all countries achieve health-related SDGs by 2030 [53].

## Additional material

Online Supplementary Document
