# Supplementary material for: Time trends in facility-based and private-sector childbirth care: analysis of Demographic and Health Surveys from 25 sub-Saharan African countries from 2000 to 2016
Source: J Glob Health. 2019 Jul 21;9(2):020406. doi: 10.7189/jogh.09.020406 (PMC6644920; doi:10.7189/jogh.09.020406)
Supplement: Online Supplementary Document [file jogh-09-020406-s001.pdf]

J Glob Health 2019; 9: 020406

Supplementary Material 1

				Sample of live births in recall period (unweighted)		T1 annual estimate of live births				T2 annual estimate of live births			
Country	T1 (2000-2007)	T2 (2008-2016)	Number of years between surveys	T1	T2	Year of estimate	Total population (thousands)	Crude birth rate (per 1,000 population)	Estimated number of births in year	Year of estimate	Total population (thousands)	Crude birth rate (per 1,000 population)	Estimated number of births in year
Benin	2001	2011	10	5,324	13,361	1999	6,664	43.4	289,222	2009	8,945	40.0	357,788
Burkina Faso	2003	2010	7	10,633	15,008	2001	11,945	41.5	495,151	2008	14,690	40.0	588,177
Cameroon	2004	2011	7	8,092	11,731	2002	16,085	41.0	659,947	2009	19,433	40.2	780,838
Chad	2004	2015	11	5,635	18,493	2002	9,002	50.2	452,263	2013	13,134	45.2	593,704
Rep of the Congo	2005	2011-12	7	4,835	9,077	2003	3,503	39.4	137,845	2009	4,254	38.8	165,214
Dem Rep of Congo	2007	2013-14	7	8,862	18,637	2005	54,751	45.4	2,484,293	2011	66,714	44.0	2,935,999
Ethiopia	2000	2016	16	10,860	10,641	1998	62,794	41.4	2,597,668	2014	97,367	33.6	3,275,613
Gabon	2000	2012	12	4,405	6,067	1998	1,171	34.4	40,335	2010	1,640	31.3	51,393
Ghana	2003	2014	11	3,820	5,882	2001	19,422	34.6	672,123	2012	25,733	32.7	841,960
Guinea	2005	2012	7	6,330	6,994	2003	9,310	41.8	388,882	2010	10,794	37.5	404,565
Kenya	2003	2014	11	5,930	20,850	2001	32,321	39.0	1,259,730	2012	43,647	33.1	1,445,707
Lesotho	2004	2014	10	3,667	3,136	2002	1,902	30.1	57,241	2012	2,090	28.7	59,908
Liberia	2007	2013	6	5,708	7,590	2005	3,261	38.6	125,994	2011	4,070	35.8	145,871
Malawi	2000	2016	16	11,886	17,285	1998	10,705	45.3	485,171	2014	17,069	38.1	650,306
Mali	2001	2012-3	12	12,965	10,326	1999	10,664	48.7	519,195	2010	15,075	44.7	674,429
Mozambique	2003	2011	8	10,294	11,102	2001	18,589	44.4	825,861	2009	23,524	42.5	1,000,549
Namibia	2000	2013	13	3,939	5,027	1998	1,811	33.3	60,223	2011	2,216	30.4	67,246
Niger	2006	2012	6	9,124	12,520	2004	13,127	52.7	691,964	2010	16,426	49.2	808,418
Nigeria	2003	2013	10	5,972	31,170	2001	125,463	43.0	5,388,905	2011	162,877	40.5	6,600,919
Rwanda	2000	2015	15	7,902	7,853	1998	7,060	41.8	295,030	2013	11,065	33.5	370,793
Senegal	2005	2015	10	10,896	6,935	2003	10,671	38.8	413,608	2013	14,120	37.6	530,712
Tanzania	2004-05	2015	11	8,553	10,232	2002	36,106	42.2	1,522,149	2013	50,637	39.8	2,015,134
Uganda	2000-01	2016	16	7,072	15,522	1998	22,552	49.3	1,112,570	2014	38,833	43.9	1,703,424
Zambia	2001	2013-4	13	6,858	13,413	1999	12,065	45.1	544,267	2011	14,265	39.3	560,519
Zimbabwe	2005-6	2015	10	5,236	6,132	2003	12,634	33.7	425,699	2013	14,387	35.6	512,582

Supplementary Material 2. Absolute number of births (annual, for midpoint of survey recall period)

	Facility deliveries					Private facility deliveries				
**Country**	**T1**	**T2**	**Absolute difference T2-T1**	**Relative change (T2-T1)/T1**	**Compound annual growth rate (T1->T2)**	**T1**	**T2**	**Absolute difference T2-T1**	**Relative change (T2-T1)/T1**	**Compound annual growth rate (T1->T2)**
Benin	221,052	311,812	90,760	41.1%	3.5%	32,624	42,863	10,239	31.4%	2.8%
Burkina Faso	190,683	391,137	200,455	105.1%	10.8%	4,159	5,882	1,722	41.4%	5.1%
Cameroon	391,216	477,717	86,500	22.1%	2.9%	117,207	164,054	46,848	40.0%	4.9%
Chad	59,834	129,843	70,009	117.0%	7.3%	8,864	6,531	-2,334	-26.3%	-2.7%
Rep of the Congo	113,295	151,254	37,959	33.5%	4.2%	9,870	19,165	9,295	94.2%	9.9%
Dem Rep of Congo	1,764,594	2,355,845	591,252	33.5%	4.2%	508,286	453,025	-55,262	-10.9%	-1.6%
Ethiopia	130,403	858,211	727,808	558.1%	12.5%	8,572	45,859	37,286	435.0%	11.1%
Gabon	34,155	46,372	12,216	35.8%	2.6%	6,195	11,461	5,265	85.0%	5.3%
Ghana	308,706	615,304	306,598	99.3%	6.5%	63,448	68,199	4,750	7.5%	0.7%
Guinea	120,281	164,092	43,811	36.4%	4.5%	5,833	19,015	13,181	226.0%	18.4%
Kenya	506,915	888,532	381,616	75.3%	5.2%	176,740	220,326	43,586	24.7%	2.0%
Lesotho	30,309	45,829	15,521	51.2%	4.2%	8,311	10,951	2,640	31.8%	2.8%
Liberia	47,071	81,556	34,485	73.3%	9.6%	12,688	18,190	5,502	43.4%	6.2%
Malawi	269,318	594,379	325,061	120.7%	5.1%	73,795	82,589	8,794	11.9%	0.7%
Mali	197,969	371,071	173,102	87.4%	5.4%	4,880	16,186	11,306	231.7%	10.5%
Mozambique	419,538	548,401	128,863	30.7%	3.4%	26,675	21,212	-5,464	-20.5%	-2.8%
Namibia	45,438	58,982	13,543	29.8%	2.0%	2,921	3,490	569	19.5%	1.4%
Niger	119,987	242,364	122,377	102.0%	12.4%	3,045	4,851	1,806	59.3%	8.1%
Nigeria	1,767,022	2,386,232	619,210	35.0%	3.0%	780,314	881,223	100,909	12.9%	1.2%
Rwanda	78,478	336,272	257,794	328.5%	10.2%	6,727	2,781	-3,946	-58.7%	-5.7%
Senegal	257,057	395,381	138,324	53.8%	4.4%	16,627	19,583	2,956	17.8%	1.6%
Tanzania	717,693	1,261,071	543,378	75.7%	5.3%	141,560	241,010	99,450	70.3%	5.0%
Uganda	409,648	1,250,313	840,665	205.2%	7.2%	160,878	274,251	113,374	70.5%	3.4%
Zambia	238,226	379,079	140,853	59.1%	3.6%	49,692	26,737	-22,955	-46.2%	-4.7%
Zimbabwe	289,475	394,637	105,161	36.3%	3.1%	53,340	61,151	7,811	14.6%	1.4%
